# Effect of Carcass Feeds on Feeding Behavior and Social Interactions in Zoo‐Based African Wild Dogs (*Lycaon pictus*)

**DOI:** 10.1002/zoo.21895

**Published:** 2025-03-12

**Authors:** Neil R. Jordan, Emily Therese Boyd, Jennifer Conaghan, Jordan Michelmore, Michelle E. Shaw, Benjamin J. Pitcher

**Affiliations:** ^1^ Centre for Ecosystem Science, School of Biological, Earth and Environmental Sciences University of New South Wales (UNSW) Sydney New South Wales Australia; ^2^ Taronga Institute of Science and Learning Taronga Conservation Society Australia Dubbo and Sydney New South Wales Australia; ^3^ School of Natural Sciences Macquarie University Sydney New South Wales Australia

**Keywords:** carnivore, dominance, feeding, husbandry, *Lycaon pictus*, social, zoo‐based animal management

## Abstract

Management of African wild dogs (*Lycaon pictus*) in zoos involves several challenges, including the difficulty of providing appropriate stimulation and enrichment for naturally wide‐ranging, energetic, cursorial hunters. Perhaps consequently, zoo packs can exhibit bouts of extreme intra‐pack aggression rarely seen in the wild. As with other species, considerable efforts are required to balance the retention and exhibition of wild‐type behaviors, against ensuring that the nutritional and welfare needs of individual group‐living animals are met. While some behaviors, such as hunting and wide‐ranging movements are impossible to mimic in zoos, the provision of food may be refined to allow natural feeding behavior to be displayed. We conducted a feeding experiment on a breeding pack of nine African wild dogs at Taronga Western Plains Zoo in Australia, presenting food in three treatments (individual pieces, butchered carcasses, and whole carcasses) to determine whether: (1) natural age‐related patterns of feeding behavior were observed; (2) food type or presentation affected feeding behavior, duration, and interactions. Free‐ranging African wild dogs exhibit an age‐based feeding structure at kill sites that is rare in other species. We found that carcass and butchered carcass feeds more closely exhibited the age‐based feeding observed in the wild. The pack spent twenty times as long consuming carcasses than food presented as individual pieces, with consumption times matching those in the wild. Carcass and butchered carcass feeds also increased the number and rate of interactions over food compared to individual pieces, with a high proportion of interactions resulting in sharing outcomes. This suggests that carcass feeds allow the exhibition of natural patterns of behavior without increasing the risk of negative social interactions. Our results highlight the importance and possibility of managing socially complex carnivores through husbandry that balances the display of natural behavior with positive animal welfare.

## Graphical abstract

Feeding carcasses to African wild dogs stimulates more natural feeding patterns and social interactions than feeding with individual pieces or butchered carcasses. Image credit: Jordan Michelmore.

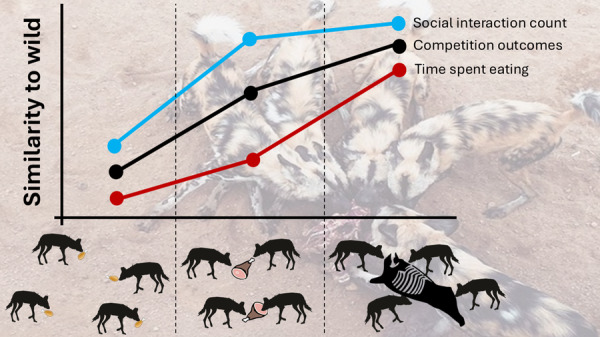

## 1 Introduction

Zoo‐based animals fulfill a key educational role as species' ambassadors, but to perform this role effectively they should display behaviors representative of their wild counterparts (Wolfensohn et al. 2018). Engaging in wild‐type behaviors can also promote learning and increase social dynamics and bonding in zoo‐based group‐living species (Shepherdson 1994). Considerable effort is required, therefore to ensure that enclosures and husbandry regimes promote natural behaviors (Newberry 1995), and this must be achieved alongside the parallel and sometimes competing goals of providing positive welfare and visitor experience.

Highly social species present several specific challenges with regard to husbandry practices. While social and enrichment requirements may be met in socially‐housed groups, the ability to display some behaviors facilitating social cohesion may be difficult to accommodate in a zoo setting. For example, while low‐ranking animals may choose to spend considerable time out of sight, sound or smell of more dominant group mates in the wild, enclosure designs may prevent this natural escape behavior in captivity, potentially resulting in elevated psychosocial stress and associated behaviors (Plowman et al. 2005), and even increased aggression and injury. Dominant female meerkats (*Suricata suricatta*), for example, engage in severe bouts of tail‐biting and dominance assertion on subordinate female group‐mates (Clutton‐Brock et al. 2008). This is a natural behavior in the wild where the targets of aggression respond by temporarily leaving the group, returning only and soon after the dominant female has given birth (Young et al. 2006). In managed settings, such temporary emigration must be physically facilitated by keepers, which may not be logistically possible, resulting in the potential for poor welfare outcomes.

Group‐living species can also present challenges for institutions to ensure that the nutritional needs of individual animals are met (Hile 2004), particularly when food is presented in more natural intact states (i.e. as whole items or carcasses). This is because more dominant animals can monopolize food at the expense of their group‐mates, and competition over food can lead to increased agonistic interactions, which, combined with several other concerns and considerations (Altman et al. 2005, Gaengler and Clum 2015), has led to zoos simultaneously providing individual portions to individuals within groups or distributing food in smaller chunks that cannot be monopolized by one or a few animals. In group‐living large carnivores, however, such feeding approaches fail to provide opportunities for animals to display natural feeding behavior. In such circumstances, zoos may fail to provide important opportunities for social interaction and competition within groups, which may affect social bonding and cohesion (Young 1997). Furthermore, a lack of food handling exposure could affect their suitability for subsequent release, should they form part of a conservation translocation (Reading et al. 2013). Carcass feeding has documented benefits to gastrointestinal health, psychological welfare, and even musculoskeletal development in large carnivores with non‐carcass diets shown to affect skull morphology (Bond and Lindburg 1990, Depauw et al. 2013, Whitehouse‐Tedd et al. 2015, Cooper et al. 2023). By modifying feeding regimes for nutritional and welfare reasons, institutions may also fail to fulfill the important educational function of exhibiting ambassador individuals/groups that showcase natural behaviors and inspire interest and empathy for their wild counterparts (Patrick et al. 2007). An understanding of the natural feeding patterns as well as social order and dynamics in social group‐living species is therefore key to developing husbandry practices that promote the performance and display of natural behaviors in zoos while meeting individual nutritional and welfare needs in line with the five freedoms of welfare and similar paradigms (Learmonth 2019).

African wild dogs (*Lycaon pictus*) are a model species for such study. They are an endangered (Woodroffe and Sillero‐Zubiri 2012), highly social species (Jordan et al. 2023), often described as the most social canid (Kat et al. 1996), and present substantial management challenges in zoos (Van den Berghe et al. 2019). Wild packs traverse and defend extremely large ranges ( ~ 700 km^2^; Pomilia et al. 2015), and socially, they are centered around a dominant pair that usually monopolize reproduction. Dominant animals can be recognized by their stereotypic scent‐marking rituals and resting associations (Jordan et al. 2014). As obligate co‐operative breeders (Courchamp and Macdonald 2001), nonbreeding individuals assist in pup care (Forssman et al. 2018), and larger packs have higher breeding success (McNutt and Silk 2008, Woodroffe et al. 2017), individual survival (Marneweck et al. 2022, Behr et al. 2023), and hunting success (Hubel et al. 2016), up to a point when intra‐pack competition begins to outweigh those benefits (Creel 2001).

While populations vary in their hunting behavior, several phases are common to most African wild dog hunts and have been described in detail elsewhere (Jordan et al. 2023). In summary, Wild dogs tend to depart on hunting excursions once or twice a day in the cooler periods of the day or even nocturnally, depending on light levels (Cozzi et al. 2012). They hunt collectively if not collaboratively (Hubel et al. 2016), and hunting excursions from dens or rest sites are preceded by a highly social “rally” (Walker et al. 2017) involving distinctive vocalizations and energetic rousing and greeting, which may play a role in maintaining social bonds (Lawick‐Goodall and Goodall 1971, Robbins 2000, Rütten and Fleissner 2004). Packs transition through an initial commuting phase to an active searching or coursing phase, which transitions through to prey selection and active chase phases upon prey encounter. Chases can but do not always cover substantial distances, and several kills may be made by different dogs or combinations of dogs in the same hunting attempt, and collaboration may occur in subduing prey. The importance of specific prey items in the diet varies regionally, with the most locally‐abundant medium‐large ungulate species usually forming the majority of the diet in a given area (Creel et al. 2004, Tshimologo et al. 2021), although smaller prey is also taken in some areas and circumstances (Woodroffe et al. 2007). Once large prey is captured, intraspecific competition for food plays an important role in within‐pack social dynamics (Lamprecht 1981). Wild dogs exhibit a dominance moderated age‐based feeding structure at kill sites (Jordan et al. 2022); a system they seem to share only with the dhole, *Cuon alpinus* (Venkataraman 1996). Broadly, pups are allowed to feed first, followed by the dominants, with subsequent access cascading through natal cohorts of increasing age. This ‘youngest‐feed‐first’ model is reportedly reinforced by the dominants (Malcolm and Marten 1982, Creel and Creel 1995, McNutt 1996). Monitoring and managing access to food is particularly important in managed settings, where individuals can compete aggressively, something that is little seen in the wild (Jordan et al. 2022).

While the promotion of natural behavior in zoo‐based African wild dogs has been investigated in studies focusing on social ranking development (Curto 2018) and environmental enrichment (Price 2010, Cloutier and Packard 2014), few published studies have assessed their feeding behavior (but see Veninga and Lemon 2001). Here we recorded the behavior of the Taronga Western Plains Zoo wild dog pack before and during feeding. Specifically, we assessed whether food presentation (i.e. individual pieces, butchered carcasses, or whole carcasses) affected social interactions. Further, as no studies have yet determined whether zoo‐born packs reflect the ‘youngest‐feed‐first’ model described in the wild, we characterized dyadic interactions around food, and compared feeding dynamics to that described in the wild (Jordan et al. 2022). Finally, as previous studies have observed increased activity and pack stimulation within an hour before wild hunts (Kühme 1965, Vogel et al. 2018), in our managed setting, we recorded whether elevated activity preceded feeding periods. Given the need to promote the retention and display of wild behaviors and maintain social cohesion in African wild dog groups, this study will benefit future management strategies, with the objective of optimizing husbandry practices of zoo‐based or temporarily housed wild packs for translocation.

## 2 Materials and Methods

### 2.1 Animals and Housing

Observations were undertaken on a pack of 9 (3 M, 6 F) African wild dogs at Taronga Western Plains Zoo, Dubbo, NSW, over ten weeks (late March to late May 2019). Two weeks before the study, five male offspring were transferred to another zoo from two litters (1 ×3.5yo M; 4 ×2.5yo M). Unfortunately, as this move occurred before the onset of study, any impacts on social behavior could not be assessed. Individuals were identifiable from their unique pelage patterns, with the pack comprising a breeding pair (10yo M, 7yo F) and their respective offspring from two consecutive litters born 3.5 years (2 M, 2 F) and 2.5 years (3 F) before the study (Supplementary Table 1).

**Figure 1:**
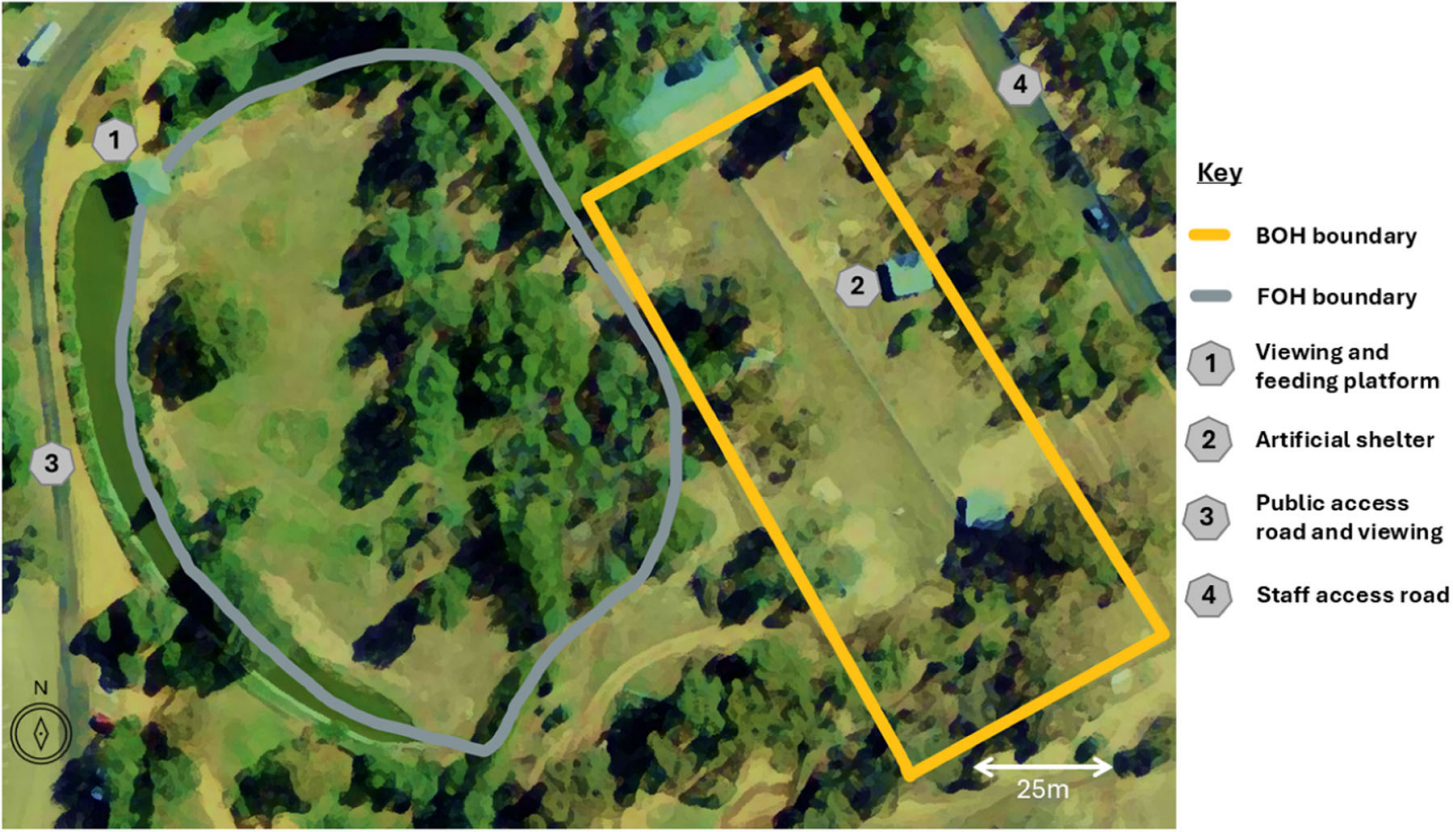
Aerial map of the African wild dog enclosure at Taronga Western Plains Zoo, showing the location and key features of the front of house (FOH) and back of house (BOH) areas.

Primary housing was in the main public enclosure at the front of house (FOH), a 9057 m^2^ open grassy yard installed with permanent den structures, vegetation, and moat separating the public from the enclosure. The group was moved from the main public exhibit to the back of house (BOH) holding area mid‐way through the study (residing there April 23 to May 24). This 5600 m^2^ BOH area was split into two equal, adjacent fenced paddocks allocated as feeding and non‐feeding enclosures, respectively. Areas are shown in Figure 1.

### 2.2 Feeding Trials and Behavioral Observations

Treatments were devised in consultation with keepers, curators, and the zoo nutritionist. Over 36 observation days, food was presented daily in one of three treatments: (Individual Portion) individual portions distributed equally to individual dogs (usually chicken meat; *N* = 14); (Butchered) rendered or butchered carcass (usually kangaroo meat and bone; *N* = 14); (Carcass) half or whole carcass (usually a kangaroo; *N* = 8). Treatments were provided in a randomized order (Supplementary Table 2). Nutritional supplements, including Calcium Carbonate powder (Limestone), Sunflower Oil and Predamax^TM^ Carnivore Supplement (Vetafarm Wagga Wagga, Australia), were coated on feeds before delivery. All feeds were delivered once daily by “yard‐toss” at 14:40 LMT from an elevated wooden viewing structure extending into the FOH or over the fence of the BOH, and in accordance with our animal ethics approval the timing and frequency of feeds followed the standard husbandry routine throughout the study. Individual portioned treatments were thrown directly to every individual, while butchered and full carcass treatments were tossed to a nonspecific region. To ensure individual‐based health and nutritional intake, extra pieces of meat were provided to individuals if they were unable to obtain a keeper‐defined amount food to support their individual predetermined energy requirements from the butchered and/or full carcass treatments. This individual provisioning is part of the routine husbandry regime for this pack, and was not altered as part of the study due to stipulations within our animal ethics approval.

Pre‐feeding and feeding behavior of individuals was recorded. FOH and BOH observations occurred over 16 days (between 26 March and 19 April) and 20 days (23 April to 24 May) respectively, with the pack being moved between these periods in advance of construction work on the FOH, and potential pregnancy of the dominant female. Data in the FOH were collected from the public viewing area. BOH observations were collected from outside a corner of the ‘feeding enclosure’.

#### 2.2.1 Pre‐Feeding Behavior

Pre‐feeding behaviors were recorded in a field notebook from visual observations. All occurrences of social rallies (sensu Walker et al. 2017) were recorded by critical incident sampling during the pre‐feeding hour. Whether the group was primarily ‘active’ or ‘inactive’ during that period was recorded by instantaneous scan sampling at 15‐min intervals preceding the feed. Data were collected using a set ethogram listing nine behaviors and pooled into ‘active’ and ‘inactive’ categories (Supplementary Table 3). Groups were classified as active or inactive according to the most prevalent behavior type during that scan.

#### 2.2.2 Feeding Behavior

Feeding behavior data was recorded using two video cameras. A stand‐alone static camera (Nikon D5100 Digital SLR) recorded a long shot of the enclosure to capture movement of pack members and distribution of food across the enclosure. One roaming hand‐held camera (Panasonic HC‐V110 HD Digital Video Camera) allowed for varying close‐ups of interactions between pack members and movement around the enclosures, aiding in the identification and tracking of individuals. Fixed camera positions were maintained in BOH and FOH throughout the study. Recording commenced once the food was thrown into the enclosure and continued until all food had been consumed or the pack had collectively ceased to engage with the provided meal for > 10 min. The last feeding was used to record the total consumption time.

Recordings from both cameras were viewed in tandem to observe activity during feeding. Type of feed/Treatment (Individual Pieces, Butchered, Carcass), and exhibit (FOH/BOH) were recorded for each feeding event. All interactions over food were recorded with individuals recorded as either “challenger” (the dog approaching) or “incumbent” (the feeding dog that was approached). Interactions over food had three outcomes, scored from the perspective of the challenger: ‘win’, ‘lose’, and ‘share’. Considering ‘sharing’ to be a socially positive, or neutral behavior, and win/lose outcomes to be more representative of feeding conflict or competition, we looked at whether conflict over food varied by Treatment type.

### 2.3 Statistical Analysis

All statistical analyses were conducted in R (R Core Team 2024). Linear Models (LMs) and Generalized Linear Mixed Models (GLMMs) utilized the *lme4* (Bates 2010) and *MuMIn* (Barton 2016) packages, and plots were produced using *ggplot2* (Wickham et al. 2016). Model selection was performed using AICc, and the terms were added stepwise, and only retained if they reduced the AICc to > 2. Results are presented from the most parsimonious models from each set. To control for repeated measures, Dyad and Day were included as random terms in all models. Dyad was a combination of the two dog IDs in the same order regardless of whether they were the incumbent or challenger in an interaction. The impact of treatment order could not be assessed in this study, as data were not collected on days before observations resulting in insufficient data on prior feeding treatment.

#### 2.3.1 Pre‐Feeding Behavior

Feeding treatment was randomized, and consequently unknown to the dogs in advance, so we could not assess whether treatment affected anticipatory behavior. Rather, we documented and described the number of rallies in the hour preceding feeding in BOH and FOH enclosures. A two‐way binomial test of proportions with continuity correction was used to assess whether enclosure affected the likelihood of a rally occurring. Similarly, to determine whether the activity of the pack increased pre‐feeding across all treatments pooled, we recorded the activity of all individuals at four 15‐min intervals in the hour before feeding using scan sampling. Descriptive statistics were used in this context.

#### 2.3.2 Feeding Behavior

We used GLMs to assess whether the duration of feeding, and the rate of dyadic interactions over food varied according to food treatment. We also scored the outcome of all dyadic interactions as win, lose, or share, from the perspective of the challenger. Share was excluded from analyses assessing whether the challenger won an outcome. Analyses were conducted using Binomial GLMMs, and these informed simple binomial tests of proportions under certain circumstances.

## 3 Results

### 3.1 Pre‐Feeding Behavior

Social rally events occurred 8 times in the hour immediately preceding feeding events and were significantly more likely to be observed in the BOH (44% of 18 sessions) than FOH (0% of 18 sessions; 2‐sample test for equality of proportions with continuity correction, X^2^_(1)_ = 7.875, *p* = 0.00501). Group‐scale activity was recorded over the hour preceding feeding, and the most frequent behavior occurring in each of four 15‐min blocks running up to feeding was recorded. Active behaviors (everything excluding resting and standing) did not increase in frequency over the hour before feeding in either enclosure, but was generally higher in the FOH than BOH across all pre‐feed periods (Table 1).

**Table 1:** Proportion of 15 min sessions in the hour preceding feeding where ‘active’ behaviors were the most frequent behavior across the group.

	Time before feeding (mins)			
Enclosure	46–60	31–45	16–30	0–15
BOH	55.56	61.11	72.22	66.67
FOH	83.33	83.33	77.78	77.78
*Pooled*	*69.44*	*72.22*	*75.00*	*72.22*

While it was rare for standing behavior to be the most prevalent activity in a session (11/144 sessions), standing increased five‐fold between the first (2.8% of sessions) and last (13.9%) 15 min of observation before feeding, suggesting anticipation of feeding.

### 3.2 Group Feeding Duration and Interaction Frequency

**Figure 2:**
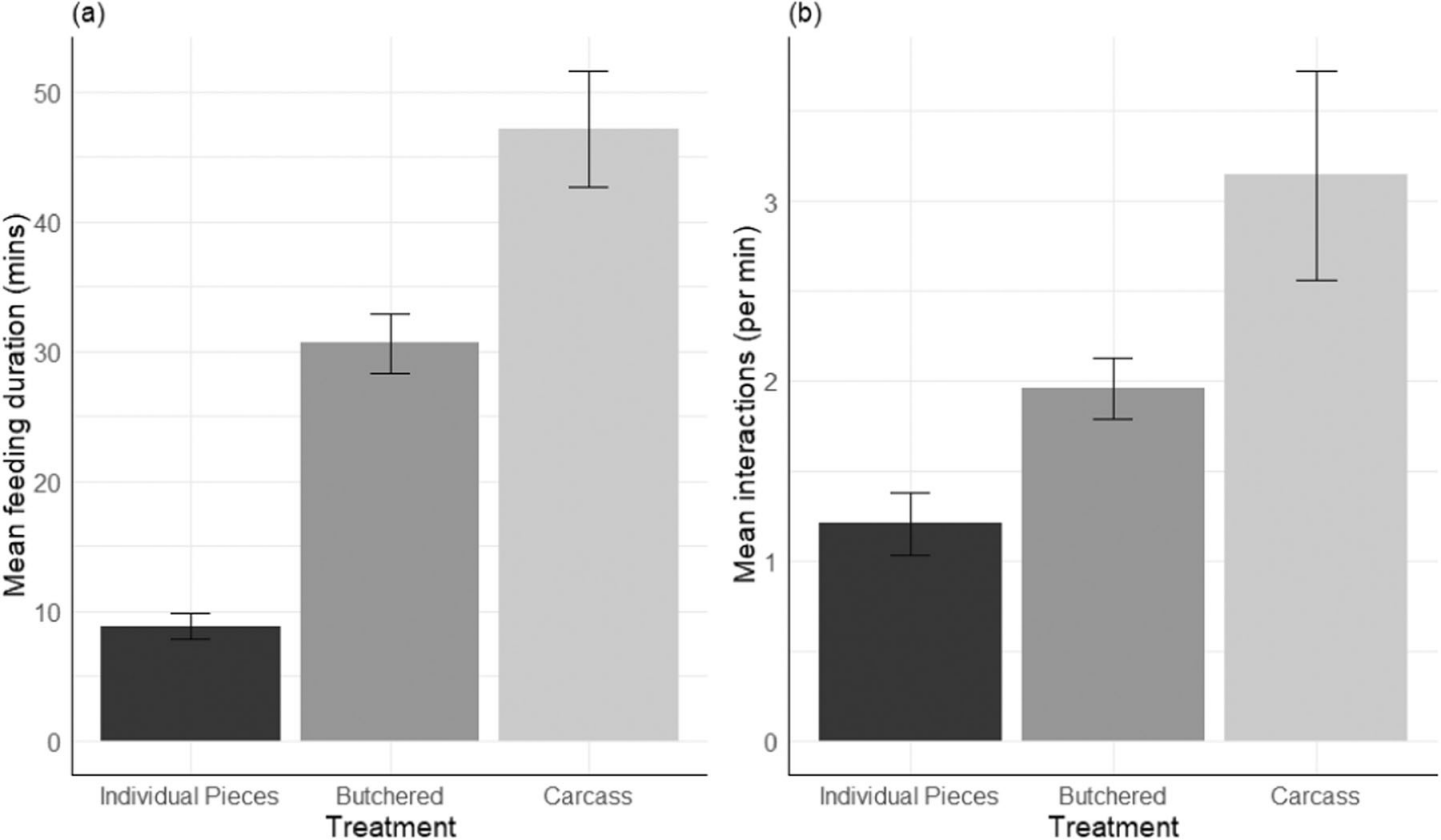
Mean feeding duration (a) and number of dyadic interactions (b) occurring during feeding events involving individual pieces of meat (*N* = 14), butchered meat (*N* = 14) and full carcass feeds (*N* = 8) to a pack of nine group‐housed African wild dogs. Error bars are 95% confidence intervals.

The total time that the group spent feeding was affected by how the food was presented (‘treatment’ hereafter). The pack spent significantly longer eating food presented as a carcass than food presented in individual pieces (Table 2; Figure 2a). While individuals tended to spend longer consuming food in the FOH area, this difference was not significant.

**Table 2:** Linear model (LM) exploring the effect of feeding method (‘Treatment’) on the duration of time a pack of 9 African wild dogs at Taronga Western Plains Zoo spent feeding. Over 36 daily feeds, food was presented as either “individual pieces” (*N* = 14), “butchered” carcasses (*N* = 14), or as a whole “carcass” (*N* = 8). Data presented here are from the most parsimonious model from a set including treatment and enclosure and their two‐way interaction. ‘Individual pieces’ was set as the reference category. All candidate models are presented in Supplementary Table 4. The top model represents the model where adding terms did not reduce the AICc by > 2.

Fixed term		Estimate	SE	t	CI (2.5%, 97.5%)	*p*
Intercept		8.846	2.207	4.008	(4.36, 13.34)	0.000329****
Treatment	(Butchered)	21.835	3.122	6.995	(15.48, 28.19)	< 0.0001***
	(Carcass)	38.351	3.660	10.477	(30.90, 45.80)	< 0.0001***

* = *p* < 0.05, **= *p* < 0.01, ***=*p* < 0.001

Overall interaction rates were also related to food treatment. Individuals interacted with one another at the highest rates (interactions/min) during a carcass feed, and at the lowest rates during individual portion feeds (Table 3; Figure 2b). Both butchered and carcass feeds had significantly higher rates of interactions than individual portion feeds. Enclosure was not retained in the models and did not significantly affect interaction rates.

**Table 3:** Linear model (LM) exploring the effect of feeding method (‘Treatment’) on the rate (/min) of dyadic interactions over food occurring in a pack of nine African wild dogs at Taronga Western Plains Zoo. Over 36 daily feeds, food was presented as either “individual pieces” (*N* = 14), “butchered” carcasses (*N* = 14), or as a whole “carcass” (*N* = 8). Data presented here are from the most parsimonious model from a set including treatment and enclosure and their two‐way interaction. ‘Individual Pieces’ was set as the reference category. All candidate models are presented in Supplementary Table 5. The top model represents the model where adding terms did not reduce the AICc by > 2.

Fixed term		Estimate	SE	t	CI (2.5%, 97.5%)	*p*
Intercept		1.2074	0.2528	4.776	(0.69, 1.72)	< 0.0001****
Treatment	(Butchered)	0.7513	0.3575	2.101	(0.024, 1.48)	0.0433*
	(Carcass)	1.9353	0.4193	4.616	(1.08, 2.79)	< 0.0001***

* = *p* < 0.05, **= *p* < 0.01, ***=*p* < 0.001.

### 3.3 Dyadic Interactions Over Food

Data were collected overall from eight carcass feeds, 14 butchered carcasses, and 14 individual portion feeds. In 2073 approaches overall, the challenger either stole the food item, failed to access the food, or joined the incumbent to feed (i.e. “shared”). Sharing outcomes were most common on carcass feeds (56.8% of 1096 approaches), and least likely on individual pieces (2.9% of 135 approaches), with butchered treatment being intermediate (39.5% of 846 approaches). Indeed, treatment affected the proportion of interactions resulting in sharing (Pearson's Chi‐squared test: *χ* ^2^_(2)_ = 166.82, *p* < 0.0001). Pairwise post‐hoc comparisons using the Bonferroni correction revealed significant differences between all treatments (butchered/pieces, carcass/pieces, butchered/carcass; all *p* < 0.0001). While this suggests that social conflict did not increase with carcass feeding, when we consider the raw number of win/lose interactions in isolation (i.e. not as proportions), butchered pieces (623) and carcasses (473) had much higher counts of win‐lose interactions compared to individual pieces (127). Overall, this suggests that conflict over food occurred more frequently at butchered pieces and carcasses than individual pieces, but interactions during these feeding events were typified by sharing outcomes.

**Figure 3:**
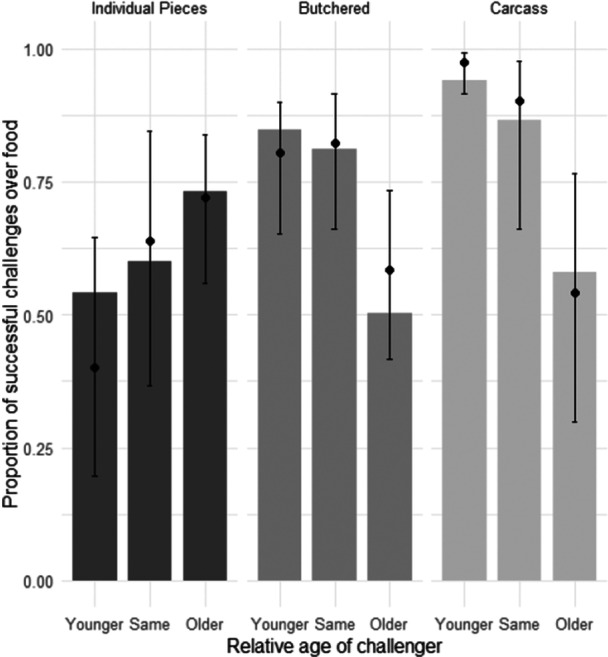
Proportion of successful challenges made over food according to the age of the challenger relative to the incumbent and the food treatment type. Bars represent actual proportions from raw data, with point and error bars representing predicted effect sizes and respective 95% confidence intervals from generalized linear mixed effects models conducted for each treatment independently and presented in Table 5. Data are based on *N* = 127, *N* = 512, *N* = 473 approaches to individuals on individual pieces of meat (*N* = 14 feeds), butchered carcasses (*N* = 14 feeds), and whole carcasses (*N* = 8 feeds) respectively.

We used a Binomial mixed effects model to identify the factors related to challengers winning encounters over food, demonstrating that challengers were significantly less likely to win if they were older than the incumbent (Table 3). Figure 3 shows that proportion of successful challenges made over food according to the age of the challenger relative to the incumbent. Despite having a limited impact on the model, treatment was also plotted, as visual exploration of the data suggested a weak trend.

We ran subsequent models assessing, separately, the likelihood of challengers winning encounters based on their relative age when contesting individual pieces, butchered carcasses, and whole carcasses. Relative age of the combatants had no impact on the outcome of dyadic contests over food for individual pieces, but was important for butchered and carcass feeds, where challengers that were older than incumbents were less likely to acquire the contested food item (Tables 4 and 5).

**Table 4:** Generalized linear mixed model (GLMM) exploring the effect of the age of the challenger compared to the incumbent of a food item. Data were collected on 1112 dyadic interactions during daily feeding events consisting of either “individual pieces” (*N* = 14), “butchered” carcasses (*N* = 14), or a whole “carcass” (*N* = 8), and involved 9 individuals in 36 dyads. Data presented here are from the most parsimonious model from a set including treatment and enclosure and their two‐way interaction. ‘Same’ age dyads were included as the reference category. All candidate models are presented in Supplementary Table 6. The top model represents the model where adding terms did not reduce the AICc by > 2. ‘Day’ and ‘Dyad’ were added as random terms at the level of the intercept to account for repeated measures.

Fixed term		Estimate	SE	z	*p*
Intercept		1.491	0.383	3.897	< 0.0001***
Relative age of challenger	Older	−1.209	0.406	−2.980	0.00289**
	Younger	0.126	0.428	0.295	0.768

* = *p* < 0.05.

** = *p* < 0.01.

*** =*p* < 0.001.

**Table 5:** Binomial mixed effects model exploring the effect of the age of the challenger compared to the incumbent in a challenger over a food item. Data were analyzed separately for dyadic interactions over (a) “individual pieces” (*N* = 127 interactions from 14 feeds, 29 dyads), (b) “butchered” carcasses (*N* = 512 interactions from 14 feeds, 34 dyads), or (c) whole “carcass” feeds (*N* = 473 interactions over 8 feeds, 34 dyads). Only the predictor 'Relative Age of challenger was incorporated into the model, and models were compared to the null with AICc. ‘Same’ age dyads were included as the reference category in each model. ‘Day’ and ‘Dyad’ were added as random terms to each model to account for repeated measures at the level of the intercept.

Fixed term		Estimate	SE	z	*p*
**(a) Individual pieces**					
Intercept		0.571	0.603	0.946	0.344
Relative age of challenger	Older	0.370	0.689	0.537	0.591
	Younger	−0.9722	0.841	−1.157	0.247
Δ *AICc to null model* = *0.419*					
**(b) Butchered**					
Intercept		1.525	0.440	3.465	0.00053***
Relative age of challenger	Older	−1.1869	0.449	2.641	−0.00826**
	Younger	−0.118	0.487	−0.243	0.808
Δ*AICc to null model* = *7.354*					
**(c) Carcass**					
Intercept		2.208	0.806	2.738	0.00618**
Relative age of challenger	Older	−2.043	0.817	−2.500	0.01243*
	Younger	1.426	0.893	1.597	0.11030
Δ *AICc to null model* = *75.468*					

* = *p* < 0.05.

** = *p* < 0.01.

*** =*p* < 0.001.

We looked at the competition dynamics within the dominant pair. In 115 dyadic interactions over food involving just the dominant pair, the dominant female was significantly more likely to challenge the dominant male than vice versa (1‐sample binomial test of proportions with continuity correction: *χ* ^2^_(1)_ = 23.513, *p* < 0.0001). Indeed, 73% of these interactions were challenges by the dominant female. The outcome of these interactions was also sex biased, with the dominant female winning a higher proportion of her challenges (83.3%) than the male did when challenging (38.7%) (2‐sample test for equality of proportions with continuity correction: *χ* ^2^_(1)_ = 19.91, *p* < 0.0001).

Finally, we investigated whether this sex‐bias in challenge and success likelihood extended to subdominant females. As we have shown that the relative age of interactants affects the outcome of challenges over food, we limited our analyses to interactions between the litter of two male and two female 3.5‐year‐old subdominants. Of 167 approaches within this cohort, a significant majority (67.1%) involved challenges by females (1‐sample proportions test with continuity correction: *χ* ^2^_(1)_ = 18.778, *p* = < 0.0001). Females were also more likely to be approached (74.3% of approaches) by litter mates than were males in this cohort (2‐sample test for equality of proportions with continuity correction: *χ* ^2^_(1)_ = 38.323, *p* = < 0.0001). While a slightly higher percentage of female incumbents lost challenges (60.5% of 124 challenges) compared to male incumbents (53.5% of 43 challenges), this difference was not significant (2‐sample test for equality of proportions with continuity correction: *χ* ^2^_(1)_ = 0.38819, *p* = 0.5333).

## 4 Discussion

A feeding experiment on a zoo‐based pack of African wild dogs demonstrated that natural age‐related patterns of feeding behavior and competition were more likely to occur when carcasses and butchered meat were presented (along with some targeted individual feeding) than when individual portions alone were provided. These treatments also affected feeding duration and interactions, with groups feeding on carcasses for longer than on food presented in other ways, and engaging in higher rates of social interactions while doing so. Furthermore, the outcomes of these dyadic interactions were more likely to result in sharing at carcasses than when smaller pieces of food were presented, and the outcome of win‐lose dyadic interactions more closely resembled the age‐based feeding patterns observed in the wild when they occurred on carcasses or butchered meat over individual pieces. These results have implications for the use of carcass feeds for environmental and social enrichment, and to facilitate the display of naturalistic behaviors in a zoo setting.

The pack spent twenty times as long consuming carcasses than food presented as individual pieces, with consumption times matching those seen in the wild (Fanshawe and Fitzgibbon 1993, Carbone et al. 2005), and previous studies on carcass feeding in zoos (Veninga and Lemon 2001). The mean carcass consumption time in the current study was 47‐min, which aligns closely with the pack studied at the same zoo previously where carcasses were consumed in 58‐min (Veninga and Lemon 2001) and with wild packs, which spend an average of 50‐min consuming a carcass (Fanshawe and Fitzgibbon 1993). While carcass feeds are considered ‘natural’ in this context, it is also important to note that although African wild dog diets are generally dominated by the most common medium‐sized prey item in a given area (Creel et al. 2004), some packs do subsist on smaller prey in the wild (Woodroffe et al. 2007). In this context, it would also be interesting to compare the competitive interactions over individual pieces provided in a zoo setting with smaller prey items captured in the wild, but competition over smaller prey has not yet been studied in the wild.

Whole carcass and butchered carcass feeds increased the number and rate of interactions over food compared to individual pieces. Some concerns regarding feeding with carcasses are that negative interactions might ensue or be more prevalent, and that some individuals might monopolize access, but neither were evident in the study. Indeed, while interactions were more common for carcass feeds, a high proportion of these resulted in sharing outcomes. As such, while interactions can increase the risk of injury and other negative impacts, a greater number of interactions in social species can have a positive impact on welfare and social behavior. That most interactions resulted in sharing outcomes was positive in this regard, but when looking at the raw frequencies of interactions, win‐lose events were still more prevalent at carcass feeds than when individuals pieces were provided. Overall however, our results concur with previous studies suggesting that carcass feeds influence activity levels and promote social dynamics and cooperation between pack members in African wild dogs (Veninga and Lemon 2001), and improve cohesion in other group‐living species (Altman et al. 2005, Gaengler and Clum 2015), collectively suggesting that carcass feeds allow natural patterns of behavior to be exhibited without substantially elevating the risk of negative social interactions. As ‘share’ outcomes have limited competition, aggression or contention, husbandry approaches may seek to promote such behavior through the increased provision of carcass feeds to invoke social interaction and strengthen pack dynamics. While it is important to recall that some individuals in this study were also target fed individual portions during carcass feeds, which may have assisted in reducing competitive interactions over food, our results highlight the possibility of managing socially complex carnivores through husbandry that balances the display of natural behavior with positive animal welfare.

Free‐ranging African wild dogs exhibit an age‐based feeding structure at kill sites that is rare in other species (Jordan et al. 2022), and shared potentially only by the dhole (Venkataraman 1996). Despite all dogs in the observed zoo‐based pack being adults (minimum age 2.5 years), thus reducing the importance of the youngest feed first model, we nevertheless found that carcass and butchered carcass feeds resulted in dogs more closely exhibiting the age‐based feeding observed in the wild. In common with wild African wild dogs, individuals were less likely to ‘win’ contested food items when they were older than the incumbent. No such effect was seen when individual pieces were fed. While previous work has suggested that the ‘youngest‐feed‐first’ model is mediated by the dominants (Malcolm and Marten 1982, Creel and Creel 1995, McNutt 1996), more recent work in the wild did not report such an effect. Consequently, we did not assess this possibility nor observe any anecdotal evidence in support of it, but suggest that future studies consider this possibility, particularly those focused on packs with pups where dominant mediation may be most common or obvious.

Promoting natural wild behavior is vital, not only during the feeding period, but also the period leading to the feeding event. In this context, social rally events, which are frenetic and involve somewhat repetitive behavior that commonly occurs before hunting excursions in the wild (Robbins 2000, Rütten and Fleissner 2004), were observed and occurred a total of eight times in the hour preceding feeding across 36 h of observations. All such events only occurred in the BOH enclosure, and were therefore not observed by the public. This frequency of rallies is relatively low compared to the wild, where wild dogs rallied 68 times across 36 sessions (Walker et al. 2017), and is perhaps surprising given the predictability of receiving food at a consistent time each type that coincides with a keeper talk to visitors, and the fact that anticipatory behavior has been linked to an animal's expectation of receiving a reward, often food or enrichment (Watters 2014). It is striking, therefore, that rallies occurred exclusively when the pack was confined to the BOH area. This area is smaller than the FOH area and so it is unexpected that this more unnaturalistic environment would promote more naturalistic behavior display. The higher density of dogs in the BOH may have necessitated the ritualized maintenance of social bonds that was less key in the larger FOH area. Indeed, while natural behaviors may be elicited in this context, the performance of these behaviors may manifest as stress‐related or relieving behavior, as occurs in pacing and other repetitive behaviors of some zoo animals (Rose et al. 2017). Repetitive predictable feeding times, coupled with low variability in feeding delivery methods may result in repetitive anticipatory behaviors (Jensen et al. 2013, Seyrling et al. 2024). Even where these behaviors are potentially natural and appropriately timed, their repetition may nevertheless be cause for concern (see Clubb and Vickery 2006). Conversely, breaks from established routines can prove stressful for zoo‐based animals, and they may exhibit ‘negative’ behaviors more frequently on ‘starve days’ (Lyons et al. 1997). In this study, the performance of natural rally behavior in possible anticipation of a forthcoming feed at levels below what occurs in the wild best aligns with reduced activity, and the lack of any increase in activity before feeding observed in this study suggests that inactivity may be the response to regular feeding times in this instance, and variation in the timing of feeds may be beneficial.

Clearly, this study is limited to a single pack, at a limited point in time, and these factors may influence the results. During part of the study ( < 3 weeks), the dominant female was in estrus, resulting in relevant behavioral changes in both the breeding pair and their offspring. The dominant female became increasingly protective of food during this time. Across the whole study, we observed that the dominant female was more likely to instigate challenges over food between the dominant pair and won a higher proportion of challenges she instigated than when the dominant male challenged her. Whether the estrus status and dominance related behaviors around this period affected this or not is not possible to decipher from our data, and as previous wild study (Jordan et al. 2022) did not assess estrus status or directly compare approaches between dominants specifically, no comparison with the wild can be made in this context either. The pack was also relocated to the BOH enclosure for the subsequent month, which may also have influenced pack dynamics and feeding priority. Indeed, some behaviors were more prevalent in the BOH exhibit, showing the importance of considering both the social and environmental context in which these behaviors occur. Additionally, five subdominant males were removed from the pack before the onset of the study. While this mimics natural dispersal in the wild, where same‐sex coalitions disperse together (McNutt 1996), this management is likely to have affected social behavior and dynamics within the pack. Unfortunately, it was not possible to assess this in this study due to transfer occurring before the study commenced, and such impacts have not previously been assessed in free‐ranging packs either. Furthermore, as it is possible that feeding competition and related social dynamics may be affected by hunger levels, which may be affected by feeding treatments presented on previous days, it is unfortunate that feeding treatment data were not collected on days before observation days. Such data should be collected in future studies. Finally, we were restricted to working on a pack without pups – the youngest individual being only 2.5 years old. Further investigations should seek to observe multiple packs with offspring ranging in ages from newborns to adults to determine if youngest‐feed‐first models are utilized by zoo‐based African wild dogs, particularly as animal care staff have anecdotally described this occurring with the same animals but in previous years when they were younger.

This study adds to the growing body of literature highlighting the important role that husbandry plays in managing socially complex carnivores in zoos, with potential application to animals held temporarily during conservation translocations. Naturalistic feeds allowed African wild dogs (and consequently the institution) to exhibit more natural behavior without increasing the propensity of negative social interactions within the pack. Through feeds that stimulate the social consumption of prey, and facilitate natural patterns of social interactions and behavior, zoos may, therefore, better showcase natural or wild‐prevalent behavior, both fulfilling their important educational role, and allowing zoo animals to better play their part as ambassadors for their wild counterparts.

## Ethics Statement

All procedures adhered to animal ethics approval 3b/02/19 issued by the Taronga Animal Ethics Committee.

## Conflicts of Interest

The authors declare no conflicts of interest.

## Supporting information

Supporting information.

## Data Availability

All data reported in the paper will be uploaded to the data repository Dryad on acceptance.
