# Supplementary material for: Effect of Carcass Feeds on Feeding Behavior and Social Interactions in Zoo‐Based African Wild Dogs (Lycaon pictus)
Source: Zoo Biol. 2025 Mar 12;44(4):313–23. doi: 10.1002/zoo.21895 (PMC12335230; doi:10.1002/zoo.21895)
Supplement: Supplementary file 1 — Supporting information. [file ZOO-44-313-s001.docx]

**Effect of carcass feeds on feeding behavior and social interactions in zoo-based African wild dogs *(Lycaon pictus)***

**Supplementary Materials**

**Supplementary Table 1.** African wild dog pack composition at Taronga Western Plains Zoo, Dubbo, Australia during the study.

| **Name** | **Sex** | **Status** | **Age (years)** |
| --- | --- | --- | --- |
| Guban* | M | Dominant | 10 |
| Kimanda* | F | Dominant | 7 |
| Patch | M | Subdominant | 3.5 |
| Chipo | M | Subdominant | 3.5 |
| Mosi | F | Subdominant | 3.5 |
| Masego | F | Subdominant | 3.5 |
| Spot | F | Subdominant | 2.5 |
| Bayana | F | Subdominant | 2.5 |
| Mambo | F | Subdominant | 2.5 |

**Supplementary Table 2.** Dates, locations (front [FOH] and back [BOH] enclosures of feeding treatments.

| **Date** | **Location** | **Treatment** |
| --- | --- | --- |
| 26/03/2019 | FOH | Individual pieces |
| 27/03/2019 | FOH | Individual pieces |
| 28/03/2019 | FOH | Carcass |
| 29/03/2019 | FOH | Butchered pieces |
| 2/04/2019 | FOH | Butchered pieces |
| 3/04/2019 | FOH | Individual pieces |
| 4/04/2019 | FOH | Carcass |
| 5/04/2019 | FOH | Butchered pieces |
| 9/04/2019 | FOH | Carcass |
| 10/04/2019 | FOH | Butchered pieces |
| 11/04/2019 | FOH | Butchered pieces |
| 12/04/2019 | FOH | Individual pieces |
| 16/04/2019 | FOH | Carcass |
| 17/04/2019 | FOH | Butchered pieces |
| 18/04/2019 | FOH | Butchered pieces |
| 19/04/2019 | FOH | Individual pieces |
| 23/04/2019 | BOH | Butchered pieces |
| 24/04/2019 | BOH | Butchered pieces |
| 25/04/2019 | BOH | Carcass |
| 26/04/2019 | BOH | Butchered pieces |
| 30/04/2019 | BOH | Individual pieces |
| 1/05/2019 | BOH | Carcass |
| 2/05/2019 | BOH | Individual pieces |
| 3/05/2019 | BOH | Butchered pieces |
| 7/05/2019 | BOH | Butchered pieces |
| 8/05/2019 | BOH | Individual pieces |
| 9/05/2019 | BOH | Individual pieces |
| 10/05/2019 | BOH | Butchered pieces |
| 14/05/2019 | BOH | Carcass |
| 15/05/2019 | BOH | Individual pieces |
| 16/05/2019 | BOH | Butchered pieces |
| 17/05/2019 | BOH | Individual pieces |
| 21/05/2019 | BOH | Individual pieces |
| 22/05/2019 | BOH | Individual pieces |
| 23/05/2019 | BOH | Carcass |
| 24/05/2019 | BOH | Individual pieces |

**Supplementary Table 3.** Definitions of behaviors of African wild dogs during pre-feeding. Data were pooled to determine if whether the group was primarily active or inactive at the time of the scan.

| **Behaviour category** | **Behaviour** | **Code** | **Definitions** |
| --- | --- | --- | --- |
| *Inactive* | *Resting* | R | Lying down. |
|  | *Standing* | S | Quadrupedal, without movement or interaction. |
| *Active* | *Walking* | W | Slow linear movement without back-tracking. |
|  | *Trotting* | Tr | Fast linear movement without back-tracking. |
|  | *Pacing* | Pa | Walking or trotting with elements of repetitive backtracking. |
|  | *Playing* | Pl | Active, non-aggressive interactions between pack members. Includes physical activities of chasing, jumping on other members or play-fighting. |
|  | *Investigating* | I | Exploring non-food object. Usually includes sniffing or biting. |
|  | *Chasing* | Ch | Actively pursuing another individual. |

**Supplementary Table 4.** Model selection table relating to Table 2 for General Linear Model (GLMs) exploring the effect of feeding method (‘Treatment’) treatment, Enclosure (BOH/FOH) and their interaction on the duration of time a pack of 9 African wild dogs at Taronga Western Plains Zoo spent feeding. Over 36 daily feeds, food was presented as either “individual pieces” (N=14), “butchered” carcasses (N=14), or as a whole “carcass” (N=8). Terms were added step-wise from the null and only retained if they reduced AICc by >2. The minimum model is represented by *, and presented in Table 2.

| **Intercept** | **Enclosure** | **Treatment** | **Enclosure:Treatment** | **df** | **LogLik** | **AICc** | **deltaAICc** | **weight** |  |
| --- | --- | --- | --- | --- | --- | --- | --- | --- | --- |
| 8.674 | + | + | + | 7 | -120.239 | 258.5 | 0.00 | 0.477 |  |
| 10.690 | + | + |  | 5 | -123.594 | 259.2 | 0.71 | 0.335 |  |
| 8.846 |  | + |  | 4 | -125.523 | 260.3 | 1.86 | 0.188 | * |
| 25.860 |  |  |  | 2 | -152.846 | 310.1 | 51.58 | 0.000 |  |
| 25.280 |  |  |  | 3 | -152.823 | 312.4 | 53.92 | 0.000 |  |

**Supplementary Table 5.** Model selection table relating to Table 3 for General Linear Model (GLMs) exploring the effect of feeding method (‘Treatment’) treatment, Enclosure (BOH/FOH) and their interaction on the rate of dyadic interactions over food (per minute) in a pack of 9 African wild dogs at Taronga Western Plains Zoo. Over 36 daily feeds, food was presented as either “individual pieces” (N=14), “butchered” carcasses (N=14), or as a whole “carcass” (N=8). Terms were added step-wise from the null and only retained if they reduced AICc by >2. The minimum model is represented by *, and presented in Table 3.

| **Intercept** | **Enclosure** | **Treatment** | **Enclosure:Treatment** | **df** | **LogLik** | **AICc** | **Delta**  **AICc** | **weight** |  |
| --- | --- | --- | --- | --- | --- | --- | --- | --- | --- |
| 1.207 |  | + |  | 4 | -47.515 | 104.3 | 0.00 | 0.767 | * |
| 1.243 | + | + |  | 5 | -47.464 | 106.9 | 2.61 | 0.208 |  |
| 1.088 | + | + | + | 7 | -46.645 | 111.3 | 6.97 | 0.024 |  |
| 1.930 |  |  |  | 2 | -56.489 | 117.3 | 13.02 | 0.001 |  |
| 1.834 |  |  |  | 3 | -56.354 | 119.5 | 15.14 | 0.000 |  |

**Supplementary Table 6.** Model selection table relating to Table 4 for Generalized Linear Mixed Model (GLMM) exploring the effect of the age of the challenger compared to the incumbent of a food item. Data were collected on 1112 dyadic interactions during daily feeding events consisting of either “individual pieces” (N=14), “butchered” carcasses (N=14), or a whole “carcass” (N=8), and involved 9 individuals in 36 dyads. The top model represents the model where adding terms did not reduce the AICc by >2. ‘Day’ and ‘Dyad’ were added as random terms to account for repeated measures. Terms were added step-wise from the null and only retained if they reduced AICc by >2. The minimum model is represented by *, and presented in Table 4.

| **Intercept** | **RelAgeChallenger** | **Treatment** | **df** | **LogLik** | **AICc** | **Delta**  **AICc** | **weight** |  |
| --- | --- | --- | --- | --- | --- | --- | --- | --- |
| 1.491 | + |  | 5 | -552.900 | 1115.8 | 0.00 | 0.872 | * |
| 1.422 | + | + | 7 | -552.760 | 1119.6 | 3.83 | 0.128 |  |
| 0.908 |  |  | 3 | -572.184 | 1150.4 | 34.60 | 0.000 |  |
| 0.723 |  | + | 5 | -571.862 | 1153.8 | 37.99 | -0.000 |  |
